# Non-alcoholic steatohepatitis-like pattern in liver biopsy of rheumatoid arthritis patients with persistent transaminitis during low-dose methotrexate treatment

**DOI:** 10.1371/journal.pone.0203084

**Published:** 2018-08-24

**Authors:** Shunsuke Mori, Nobuyuki Arima, Masahiro Ito, Shigetoshi Fujiyama, Yasuhiro Kamo, Yukitaka Ueki

**Affiliations:** 1 Department of Rheumatology, Clinical Research Center for Rheumatic Diseases, NHO Kumamoto Saishunsou National Hospital, Kohshi, Kumamoto, Japan; 2 Department of Pathology, Kumamoto Shinto General Hospital, Kumamoto, Japan; 3 Department of Pathology, Clinical Research Center, NHO Nagasaki Medical Center, Omura, Nagasaki, Japan; 4 Department of Gastroenterology and Hepatology, Kumamoto Shinto General Hospital, Kumamoto, Japan; 5 Gastrointestinal Endoscopy Center, Sasebo Chuo Hospital, Sasebo, Nagasaki, Japan; 6 Rheumatic and Collagen Disease Center, Sasebo Chuo Hospital, Sasebo, Nagasaki, Japan; Medizinische Fakultat der RWTH Aachen, GERMANY

## Abstract

**Objective:**

The mechanism of liver injury with low-dose methotrexate (MTX) is incompletely understood. This study was designed to evaluate the association between non-alcoholic fatty liver disease (NAFLD) and liver injury during MTX treatment for rheumatoid arthritis (RA).

**Methods:**

Between October 2014 and May 2015, we enrolled all MTX users for RA and monitored participant serum hepatic transaminase levels for 1 year. All patients had normal transaminase levels before the first MTX prescription. Using diagnostic criteria for non-alcoholic steatohepatitis (NASH), we performed histological analyses for patients presenting persistent transaminitis, defined as elevations of hepatic transaminases in four of six determinations during the follow-up period. Possible risk factors for persistent transaminitis were also examined.

**Results:**

We followed 846 RA patients with a mean cumulative MTX dose of 2.48 g and identified 51 patients presenting persistent transaminitis. According to multivariate logistic regression analysis, obesity (odds ratio [OR] 3.23, *p* < 0.001), type 2 diabetes (OR 3.52, *p* = 0.001), hypercholesterolemia (OR 2.56, *p* = 0.004), and hyperuricemia (OR 3.52, *p* = 0.019), which are recognized as risk factors for NAFLD, were independently associated with a risk of persistent transaminitis. Among patients with persistent transaminitis, 42 showed fatty liver at ultrasonography. These patients had no evidence of alcoholic fatty liver, chronic viral hepatitis, autoimmune liver diseases, or hereditary liver diseases. Biopsy specimens were obtained from 32 patients, and we found that a NASH-like pattern was the most prevalent histological abnormality. There was no significant impact of MTX dose and duration on the histological severity.

**Conclusion:**

Risk factors and histological findings are similar between NAFLD/NASH and liver injury during low-dose MTX treatment for RA, which suggests a strong association between both entities. NAFLD/NASH may be an underlying condition causing persistent transaminitis in MTX-treated RA patients. The results of this study illustrate the need for monitoring liver injury in RA patients with NAFLD risk factors during MTX treatment.

## Introduction

Over the last decade, the management of rheumatoid arthritis (RA) has progressed substantially through early and aggressive intervention with disease-modifying antirheumatic drugs (DMARDs) [1, 2]. Methotrexate (MTX) is currently used worldwide as the DMARD of first choice in the treatment of early and established RA, even after the advent of biological molecular-targeted agents [3–5]. With the continued use of MTX, however, hepatotoxicity has been an important safety concern in RA patients [6]. A recent systemic review of the medical literature showed that the incidence of hepatic transaminase elevations during the first 3 years of low-dose MTX use for RA was 13 per 100 patient-years, with a cumulative incidence rate of 31% [7]. Another systemic review also showed that 20% of RA patients had at least one episode of elevated liver enzymes during an average of 4.6 years of low-dose MTX treatment [8]. Nevertheless, the mode of action underlying liver injury during MTX treatment is incompletely understood, and it remains unclear whether low-dose MTX can independently contribute to liver injury in these patients.

Nonalcoholic fatty liver disease (NAFLD) is a leading cause of chronic liver disease worldwide [9–11]. Although its prevalence has been reported variously depending on the population studied and the definition used, a recent meta-analysis study indicated that the global prevalence of imaging-diagnosed NAFLD is estimated to be 25% [12]. NAFLD encompasses a large spectrum of pathological changes, ranging from simple steatosis (nonalcoholic fatty liver [NAFL]) to the more progressive form of NAFLD, namely, nonalcoholic steatohepatitis (NASH). NASH is defined in histological terms, namely, the presence of hepatic steatosis and inflammation with hepatocellular injury (ballooning degeneration) and varying degrees of fibrosis, which can progress to cirrhosis, liver failure, and hepatocellular carcinoma [9–11]. NASH patients, in particular those with advanced stages of fibrosis, have a higher risk of liver-related mortality than non-NASH NAFLD patients [12–16]. Obesity, type 2 diabetes, hyperlipidemia, hypertension, hyperuricemia, and metabolic syndrome have been recognized as risk factors for NAFLD [12, 17–19]. With the growing epidemics of these conditions, the prevalence of NAFLD is expected to increase dramatically. It is of note that several risk factors for NAFLD are also associated with liver injury in psoriasis patients receiving MTX treatment [20–23]. In addition, recent studies using liver biopsies have shown that the pathological features of liver injury during MTX treatment resembled those of NASH in psoriasis patients, suggesting that NASH can predispose or contribute to liver injury in these patients [21, 23]. Regarding the association between NASH and liver injury observed in RA patients during MTX treatment, however, there is little information available.

In this study, we performed a 1-year follow-up for MTX-treated RA patients and identified patients who developed persistent transaminitis during MTX treatment. For these patients, the grade and severity of hepatic steatosis, inflammation, cellular degeneration, and fibrosis were evaluated according to four pathological criteria for NASH diagnosis. We also examined the association between the development of persistent transaminitis and clinical features, such as RA-related factors, cumulative dose of MTX, and NAFLD risk factors.

## Patients and methods

### Patients and study design

Between October 2014 and May 2015, we consecutively enrolled all MTX users for RA at outpatient clinics for rheumatic diseases at NHO Kumamoto Saishunsou National Hospital and Sasebo Chuo Hospital in Japan. Eligible patients were required to have normal levels of alanine aminotransferase (ALT) and aspartate aminotransferase (AST) at the time of first starting MTX treatment. We confirmed that all candidate MTX users had exhibited normal levels of ALT and AST at the initiation of this drug. Patients were also required to fulfil the 1987 American College of Rheumatology (ACR) criteria or the 2010 ACR/European League Against Rheumatism (EULAR) criteria for diagnosis of RA [24, 25]. The exclusion criteria were being under 18 years of age, use of hepatotoxic drugs except RA medications, or having acute hepatic disease.

For each patient, demographic characteristics, RA-related factors, such as RA duration, Steinbrocker’s stage, clinical disease activity index (CDAI), health assessment questionnaire (HAQ), a level of serum C-reactive protein (CRP), positivity of anti-cyclic citrullinated peptide antibodies (anti-CCP Abs), the use of biological agents, steroids, nonsteroidal anti-inflammatory agents (NSAIDs), and acetaminophen, together with the presence of NAFLD risk factors and comorbidities, including hypertension, type 2 diabetes, chronic kidney disease (CKD), smoking history, body mass index (BMI), a level of serum low-density lipoprotein cholesterol (LDL-C), and level of serum uric acid, were examined at enrollment. The definitions of hypertension, diabetes, and CKD were described elsewhere [26].

To identify patients who developed persistent transaminitis, we monitored participant serum ALT and AST levels at each visit (i.e., every 4–8 weeks) for 1 year. Persistent transaminitis was defined as elevations in ALT and AST levels above the upper limit of normal (ULN) in four of six determinations during the 1-year follow-up period. If serum levels of transaminases were increased by greater than threefold the ULN, MTX was discontinued [27], but monitoring of the enzymes was continued to the end of the follow-up period. All patients who were found to have persistent transaminitis underwent abdominal ultrasonography (US), together with autoantibody tests (antinuclear antibody [ANA] and anti-mitochondrial antibody M2). ULN values for ALT and AST used in this study were 30 IU/l. Patients who presented abnormal findings suggesting fatty liver or chronic liver disease such as hepatic fibrosis and cirrhosis at US were scheduled to undergo liver biopsy. The exclusion criteria for liver biopsy in this study were the presence of secondary causes of hepatic fat accumulation, including chronic viral hepatitis (hepatitis B and hepatitis C), significant ethanol intake (> 30 g/day for males and > 20 g/day for females), autoimmune liver diseases (autoimmune hepatitis and primary biliary cholangitis), and hereditary liver diseases [9–11]. Patients who were seropositive for autoantibodies but had no pathological evidence to diagnose autoimmune liver disease were not excluded from liver biopsy because the presence of autoantibodies is reported in one-quarter of patients with NAFLD. Liver biopsy is therefore required to rule out autoimmune liver disease in NAFLD patients with positive autoantibodies [28, 29].

### Abdominal US and HRCT

Abdominal US and HRCT were performed and viewed in random order and independently by two board-certified experts in hepatology (SF and YK). Final decisions were made by consensus in the event of a disagreement. Fatty liver was diagnosed when all of the following four abnormal findings were observed on US scan: bright liver, hepatorenal contrast, vascular blurring, and deep attenuation [30]. Chronic liver disease such as fibrosis and cirrhosis was identified based on the following morphological abnormalities: irregular or nodular liver surface, coarse or non-homogeneous liver parenchymal echotexture, and blunted or rounded liver edge, [31]. HRCT findings were used to identify fatty infiltration in the liver and determine its severity. For quantitative evaluations, the liver-to-spleen attenuation ratio (L/S ratio) was calculated. Hepatic and splenic attenuation values were measured on unenhanced HRCT scans using four circular region-of-interest (ROI) cursors in the liver (two in the right lobe and two in the left lobe) and two in the spleen. Average attenuation values of the liver and spleen were calculated and used to determine the L/S ratio. We interpreted an L/S ratio < 1.1 as indicative of fatty deposition > 30% [32].

### Liver biopsy

For assessment of the type and severity of liver injury, we performed US-guided liver biopsy. Each biopsy specimen was evaluated using four pathological criteria, namely, the Matteoni criteria [33], the Brunt criteria for grading and staging [34], the NASH Clinical Research Network (NCRN) pathologic criteria (NAS: NAFLD Activity Score) [35], and the Younossi criteria [36]. First, we made a differential diagnosis between NASH and non-NASH NAFLD (NAFL) based on the following histological findings: steatosis, lobular inflammation, centrilobular ballooning degeneration of hepatocytes, Mallory-Denk bodies, centrilobular pericellular/perisinusoidal fibrosis, and bridging fibrosis. In the Matteoni criteria, types 1 and 2 were diagnosed with non-NASH NAFLD (NAFL), and types 3 and 4 were defined as NASH. Grading of disease activity and staging of fibrosis were made based on the NAS system and the Brunt criteria. In the NAS system, liver biopsy specimens were scored separately for steatosis (grades 0‒3), lobular inflammation (grades 0‒3), hepatocellular ballooning (grades 0‒2), and fibrosis (stages 0‒4). The sum of grades for steatosis, inflammation, and ballooning was used as the NAS. Fibrosis was scored as follows: stage 1, perisinusoidal or periportal fibrosis; stage 2, perisinusoidal and portal/periportal fibrosis; stage 3, bridging fibrosis; and stage 4, cirrhosis. Similarly, in the Brunt criteria, the grading of NASH activity was performed according to the degree of steatosis, ballooning degeneration, lobular and portal inflammation (grades 1‒3), and the staging of fibrosis was categorized as follows: stage 1, zone 3 perisinusoidal/pericellular fibrosis; stage 2, the above pattern plus periportal fibrosis; stage 3, the above two patterns plus bridging fibrosis; stage 4, cirrhosis. Patients’ liver biopsies were reviewed in random order and independently by two board-certified experts in liver pathology (NA and MI). Both observers were blinded to the patients’ clinical status. Final diagnosis was determined by consensus if there was a disagreement between their interpretations.

### History of RA medications

Cumulative doses of MTX and steroids (prednisolone equivalent) at the time of enrollment as well as at the time of liver biopsy were calculated for each user. In the present study, 85% of patients were first diagnosed with RA in our institutions and started MTX treatment. Information on weekly MTX dose and duration of treatment was collected from each division’s computer database. The remaining 15% of patients started MTX treatment at other hospitals, and we scrutinized weekly MTX dose and duration from data-providing documents from each patient’s treating doctor in these hospitals. We confirmed that 5 mg/week of folic acid was prescribed concomitantly during MTX therapy.

### Ethics approval

This study was conducted in accordance with the principles of the Declaration of Helsinki (2008). The protocol of this study also meets the requirements of the Ethical Guidelines for Medical and Health Research Involving Human Subjects, Japan (2014), and has been approved by the Human Research Ethics Committee of NHO Kumamoto Saishunsou National Hospital (No. 26–04). Informed written consent was obtained from all participants.

### Statistical analysis

In univariate analyses for comparisons of categorical variables, differences between patient groups were analyzed using the chi-square test or Fisher’s exact probability test. Continuous variables were assessed by the independent-measures *t*-test for comparisons between two patient groups and analysis of variance (ANOVA) with a post hoc Tukey’s honest significant difference (HSD) test for comparisons between three patient groups. Multivariate logistic regression analysis was performed to evaluate the association between persistent transaminitis as a dependent variable and a set of independent variables considered to be significant risk factors in univariate analyses. A backward stepwise selection procedure was used to select significant independent variables. The strength of association between persistent transaminitis and these independent variables was estimated using odds ratios (ORs) and 95% confidence intervals (95% CIs). In addition, the receiver operating characteristic (ROC) curve and the corresponding area under the curve (AUC) value were calculated to provide an index of validity for the multivariate logistic regression model. For all tests, probability values (*p* values) < 0.05 were considered to indicate statistical significance. All calculations were performed using PASW Statistics version 22 (SPSS Japan Inc., Tokyo, Japan).

## Results

### Comparisons of clinical characteristics between RA patients with and without presenting persistent transaminitis during the 1-year follow-up period

A total of 846 MTX users for RA (mean cumulative dose of 2.48 g) were consecutively enrolled in this study and monitored their serum ALT and AST levels for 1 year. All patients had received MTX treatment for at least 1 month at the time of enrollment. Among these patients, persistent transaminitis was found in 51 patients. Nine of the 51 patients discontinued MTX treatment during the follow-up period because of an increase in ALT/AST levels > 3 times the ULN, but enzyme levels continued to be above the ULN. As shown in Table 1, age (59.0 vs. 63.8 years, *p* = 0.007), RA duration (8.3 vs. 11.3 years, *p* = 0.02), obesity (BMI ≥ 25, 45.1 vs. 19.7%, *p* < 0.001), type 2 diabetes (25.5 vs. 10.1%, *p* = 0.002), hypercholesterolemia (LDL-C levels ≥ 140 mg/dl, 33.3 vs. 17.1%, *p* = 0.007), and serum uric acid levels (5.1 vs. 4.6 mg/dl, *p* = 0.01) were significantly different between patients with persistent transaminitis and those without. Rates of CKD, hypertension, and current/ex-smokers were similar between both groups. In addition, there was no significant association of persistent transaminitis with sex or RA-related indexes, including RA stage, CDAI, HAQ, serum CRP levels, or anti-CCP positivity.

**Table 1:** Baseline characteristics of MTX users for RA who did or did not develop persistent transaminitis during follow-up.

	Total (n = 846)	With transaminitis* (n = 51)	Without transaminitis* (n = 795)	*p*^†^
Age, years, mean (SD)	63.5 (12.2)	59.0 (7.9)	63.8 (12.3)	0.007
≥ 65 years, number (%)	421 (49.8)	14 (27.5)	407 (51.2)	0.001
Male/female, number	162/684	14/37	148/647	0.14
RA duration, years, mean (SD)	11.1 (9.0)	8.3 (7.0)	11.3 (9.0)	0.02
Anti-CCP (+), number (%)	710 (83.9)	43 (84.3)	667 (83.9)	1.00
Stage III/IV, number (%)	415 (49.1)	18 (35.3)	397 (49.9)	0.05
CDAI, mean (SD)	6.0 (7.6)	7.7 (9.7)	5.9 (7.3)	0.12
HAQ, mean (SD)	0.30 (0.50)	0.28 (0.51)	0.30 (0.50)	0.83
Serum CRP, mg/dl, mean (SD)	0.27 (0.76)	0.48 (0.92)	0.26 (0.74)	0.05
BMI, kg/m^2^, mean (SD)	22.6 (3.6)	25.8 (5.2)	22.4 (3.4)	< 0.001
Obesity (≥ 25), number (%)	180 (21.3)	23 (45.1)	157 (19.7)	< 0.001
Hypertension, number (%)	306 (36.2)	21 (41.2)	285 (35.8)	0.46
Type 2 diabetes, number (%)	93 (11.0)	13 (25.5)	80 (10.1)	0.002
Serum LDL-C, mg/dl, mean (SD)	114.0 (31.2)	127.1 (38.9)	113.2 (30.5)	0.002
≥ 140 mg/dl, number (%)	153 (18.1)	17 (33.3)	136 (17.1)	0.007
Serum uric acid, mg/dl, mean (SD)	4.6 (1.3)	5.1 (1.6)	4.6 (1.3)	0.01
> 7.0 mg/dl, number (%)	46 (5.4)	5 (9.8)	41 (5.2)	0.19
eGFR, ml/min/1.73 m^2^, mean (SD)	76.0 (20.2)	77.0 (17.4)	76.0 (20.4)	0.74
CKD, number (%)	178 (21.0)	10 (19.6)	168 (21.1)	1.00
Current/ex-smokers^‡^, number (%)	203 (24.0)	14 (27.5)	189 (23.8)	0.61
Use of biologics, number (%)	365 (43.1)	24 (47.1)	341 (42.9)	0.56
Use of NSAIDs, number (%)	178 (21.0)	8 (15.7)	170 (21.4)	0.38
Cumulative MTX dose, g, mean (SD)	2.48 (2.17)	2.37 (1.56)	2.49 (2.20)	0.71
Use of steroids, number (%)	179 (21.2)	10 (19.6)	169 (21.3)	0.86
Cumulative dose, g, mean (SD)	4.96 (5.56)	2.29 (1.27)	5.12 (5.67)	0.12

All data were determined at the time of enrollment in this study.

*Defined as elevations in ALT and/or AST levels above the upper limit of normal in four of six determinations during a 1-year follow-up period.

^†^Comparison between patients with persistent transaminitis and those without this complication.

^‡^Defined as current or former smokers with a smoking history ≥ 10 pack-years.

RA, rheumatoid arthritis; ALT, alanine aminotransferase; AST, aspartate aminotransferase; anti-CCP, anti-citrullinated peptide antibodies; CDAI, clinical disease activity index; HAQ, health assessment questionnaire; CRP, C-reactive protein; BMI, body mass index; LDL-C, low-density lipoprotein cholesterol; eGFR, estimated glomerular filtration rate; NSAIDs, nonsteroidal anti-inflammatory drugs; MTX, methotrexate; SD, standard deviation

In terms of RA treatment, the mean cumulative dose of MTX was not significantly different between patient groups. In addition to MTX, 43.1% of patients were receiving a biological agent. Steroids and NSAIDs were used in 21.2% and 21.0% of patients, respectively. There were no significant differences in the rates of prescription of these drugs between the two groups. In addition, there was no significant difference in the mean cumulative dose of steroids between both groups. Acetaminophen was not prescribed to any patients.

The clinical and therapeutic characteristics of nine patients who discontinued MTX due to an increase in ALT/AST > 3 times the ULN are summarized in Table 2. All patients except case 9 had at least one of the following conditions: obesity, type 2 diabetes, hyperlipidemia, and hyperuricemia. Cumulative MTX dose and treatment duration varied among patients.

**Table 2:** Summary of details from MTX users for RA who exhibited ALT/AST levels > 3 times the ULN during a 1-year follow-up period.

Case	Age/sex	RA duration (years)	RA stage	BMI	LDL-C	Uric acid	Type 2 diabetes	Cumulative MTX dose (g)	MTX duration (months)	Liver biopsy
				≥ 25	≥ 140 mg/dl	≥ 7.0 mg/dl				
1	63F	18	III	No	Yes	No	Yes	0.2	6	NASH
2	77F	2	III	No	No	Yes	Yes	0.3	11	NASH
3	76F	4.1	IV	No	Yes	No	No	4.6	6	NASH
4	57F	17	III	Yes	Yes	No	No	5.6	13	NAFL
5	62M	2.2	I	Yes	No	No	Yes	0.3	9	NASH
6	63F	3	I	Yes	Yes	No	No	0.7	16	NASH
7	59F	1.5	I	No	Yes	No	No	0.3	7	NASH
8	60F	8	IV	Yes	Yes	No	No	3.3	96	NASH
9	62F	15	I	No	No	No	No	1.0	60	NASH

All data, except for histology, were determined at the time of enrollment in this study.

RA, rheumatoid arthritis; ALT, alanine aminotransferase; AST, aspartate aminotransferase; BMI, body mass index; LDL-C, low-density lipoprotein cholesterol; MTX, methotrexate; NASH, nonalcoholic steatohepatitis; NAFL, nonalcoholic fatty liver; ULN, upper limit of normal

### Factors associated with the development of persistent transaminitis during MTX treatment for RA

Advanced age (≥ 65 years), RA duration, obesity, type 2 diabetes, hypercholesterolemia, and hyperuricemia were included, as independent variables, in multivariate logistic regression analysis. As shown in Table 3, obesity (OR 3.23, *p* < 0.001), type 2 diabetes (OR 3.52, *p* = 0.001), hypercholesterolemia (OR 2.56, *p* = 0.004), and hyperuricemia (OR 3.52, *p* = 0.019) were significant factors associated with the development of persistent transaminitis during MTX treatment for RA. Advanced age was negatively associated with this abnormality (OR 0.29, *p* < 0.001). The final model yielded an AUC-ROC of 0.74 (p < 0.001).

**Table 3:** Factors associated with the development of persistent transaminitis during MTX treatment for RA.

	OR	95% CI	*p*
Age, ≥ 65 years (vs. < 65 years)	0.29	0.15‒0.56	< 0.001
BMI, ≥ 25 (vs. < 25)	3.23	1.76‒5.92	< 0.001
Type 2 diabetes, yes (vs. no)	3.52	1.69‒7.31	0.001
Serum LDL-C, ≥ 140 mg/dl (vs. < 140 mg/dl)	2.56	1.34–4.89	0.004
Serum uric acid, > 7.0 mg/dl (vs. ≤ 7.0 mg/dl)	3.52	1.23‒10.12	0.019

Multivariate logistic regression analysis was conducted to evaluate factors associated with the development of persistent transaminitis during MTX treatment for RA. Independent factors that remained in the final model are shown. The final step yielded an AUC-ROC of 0.74 (95% CI 0.67‒0.81, *p* < 0.001).

RA, rheumatoid arthritis; BMI, body mass index; LDL-C, low-density lipoprotein cholesterol; MTX, methotrexate; OR, odds ratio; 95% CI, 95% confidence interval; AUC, area under the curve; ROC, receiver operating characteristics

### Histological patterns in RA patients with persistent transaminitis

**Fig 1:**
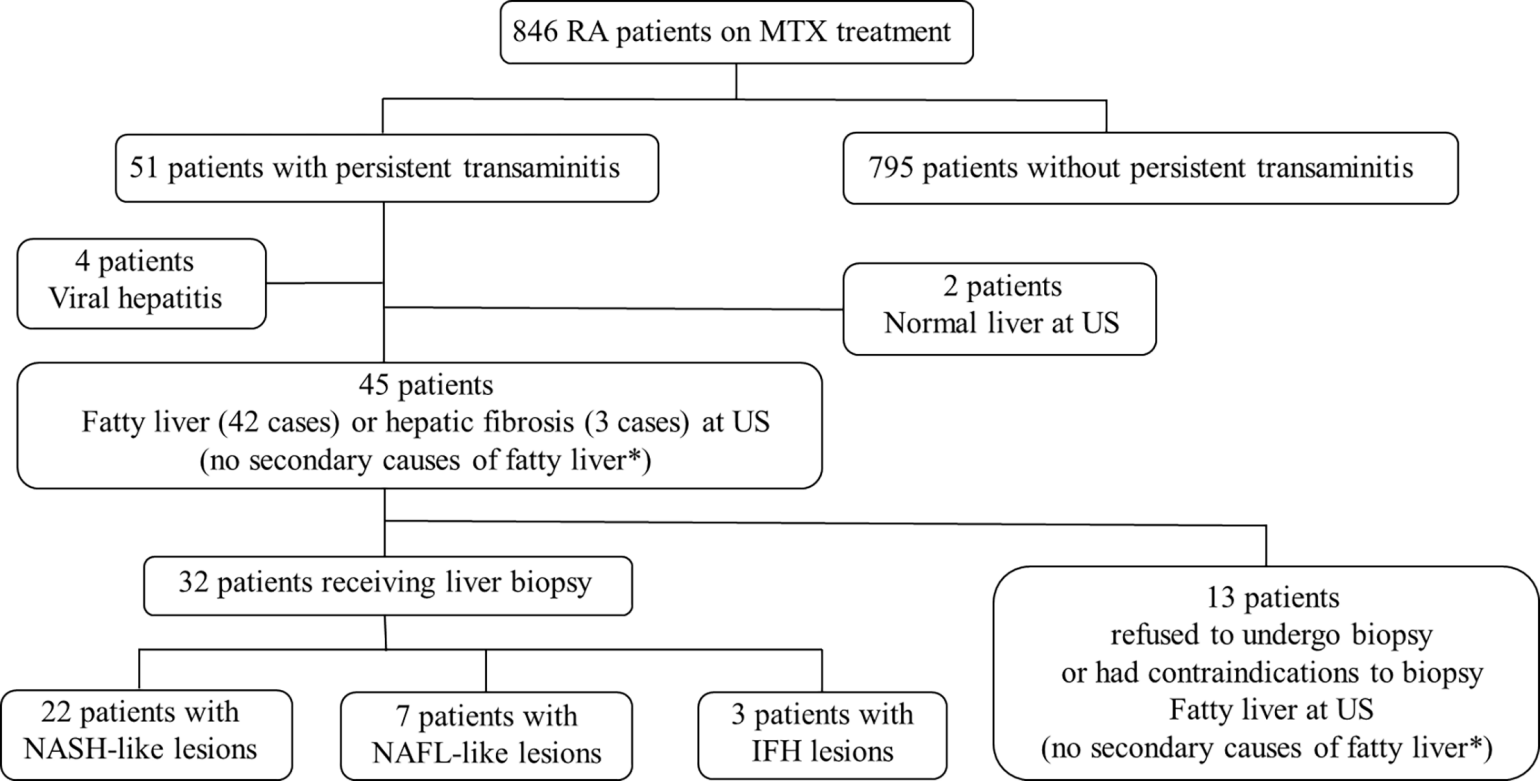
Flowchart for examinations of persistent transaminitis observed during MTX treatment for RA. *Secondary causes of fatty liver include significant ethanol intake (> 30 g/day for male and > 20 g/day for female), viral hepatitis (hepatitis B and hepatitis C), autoimmune liver disease (autoimmune hepatitis and primary biliary cholangitis), and hereditary liver diseases. RA, rheumatoid arthritis; MTX, methotrexate; NASH, non-alcoholic steatohepatitis; NAFL, non-alcoholic fatty liver; IFH, interface hepatitis; US, ultrasonography; HRCT, high-resolution computed tomography.

Fig 1 shows a flowchart for the evaluation of persistent transaminitis observed during the follow-up period. Of 51 patients with persistent transaminitis, 4 were diagnosed with viral infection (hepatitis B or C), and 2 did not show abnormal US findings. The remaining 45 patients showed fatty liver (42 patients) or hepatic fibrosis (3 patients) at US examinations, but they did not have any evidence of alcoholic fatty liver, chronic viral hepatitis, autoimmune liver diseases or hereditary liver diseases. In addition, none of the 45 patients showed symptoms or laboratory abnormalities that would lead us to suspect a diagnosis of endocrine disorders. Among these patients, 1 had a contraindication to liver biopsy and 12 refused to undergo this procedure. The mean L/S ratio (standard error) on the HRCT scans of the 13 patients was 0.99 (0.04), suggesting the presence of fatty liver with deposition > 30% in these patients.

The remaining 32 patients underwent liver biopsy (Fig 1). Histological findings of these patients are shown in Table 4. Among them, 22 patients were found to have steatosis, centrilobular ballooning degeneration, and centrilobular perisinusoidal fibrosis. Based on these findings, they were classified as Matteoni criteria type 4. We diagnosed these patients as having a NASH-like pattern (NASH-like patients). Histological findings of the other 10 patients did not meet the Matteoni criteria for NASH. Seven patients had steatosis without lobular inflammation or centrilobular ballooning, and they were classified as Matteoni criteria type 1. These patients were diagnosed as having a NAFL-like pattern (NAFL-like patients). The histological diagnosis of the remaining 3 patients was interface hepatitis. Among the patients who underwent liver biopsy, advanced stage of fibrosis (stage ≥ 3) was found in 15 patients (bridging fibrosis), but cirrhosis was not detected in any cases. Among 9 patients whose serum transaminase levels were increased by greater than threefold the ULN, 8 had a NASH-like pattern and 1 had a NAFL-like pattern (Table 2).

**Table 4:** Characteristics of RA patients who underwent liver biopsy for histological assessment of liver injury during MTX treatment.

	NASH-like lesions	NAFL-like lesions	IFH lesions	p*
	(n = 22)	(n = 7)	(n = 3)	
Age, years, mean (SE)	60.4 (1.5)	60.0 (3.6)	50.3 (3.7)	0.99
RA duration, years, mean (SE)	9.2 (1.7)	9.2 (2.5)	13.5 (2.1)	1.00
HRCT findings				
L/S ratio at biopsy, mean (SE)	0.96 (0.04)	1.19 (0.08)	1.29 (0.03)	0.018
L/S ratio before MTX therapy, mean (SE)	0.97 (0.08) (n = 14)	1.18 (0.04) (n = 4)	1.22 (n = 1)	0.10
Pathological findings				
Steatosis	22 (100)	7 (100)	0	-
Lobular inflammation, number (%)	21 (95.5)	0	2 (66.7)	-
Centrilobular ballooning, number (%)	22 (100)	0	0	-
Centrilobular fibrosis, number (%)	22 (100)	1 (14.3)	1 (33.2)	-
Bridging fibrosis, number (%)	13 (59.1)	1 (14.3)	1 (33.3)	-
Cirrhosis, number (%)	0	0	0	-
Grade of NAS element/Stage of fibrosis				
Steatosis grade ≥ 2, number (%)	9 (40.9)	0	0	-
Inflammation grade ≥ 2, number (%)	7 (31.8)	0	1 (33.2)	-
Ballooning grade 2, number (%)	5 (22.7)	0	0	-
NAS ≥ 3, number (%)	19 (86.4)	0	0	-
NAS, mean (SE)	3.9 (0.3)	0.1 (0.1)	1.0 (0.6)	-
Fibrosis stage ≥ 3, number (%)	13 (59.1)	1 (14.3)	1 (33.3)	-
Positive autoantibodies				
Antinuclear antibody, number (%)	16 (72.7)	4 (57.1)	2 (66.7)	0.64
Anti-mitochondrial M2, number (%)	1 (4.5)	0	0	1.00
Hepatic transaminases				
AST, IU/l, mean (SE)	51.0 (3.8)	50.3 (9.4)	45.0 (12.1)	1.00
ALT, IU/l, mean (SE)	64.4 (4.7)	47.0 (5.0)	47.3 (5.9)	0.13
MTX use at the time of liver biopsy				
Duration, months, mean (SE)	46.5 (12.0)	47.3 (19.0)	90.7 (22.3)	0.42
Cumulative dose, g, mean (SE)	2.34 (0.35)	2.39 (0.58)	4.54 (0.70)	0.09
Use of steroids, number (%)	4 (18.2)	1 (14.3)	0	1.00
Cumulative dose, g, mean (SD)	1.20 (1.18)	1.00	-	0.50

Data were obtained at the time of liver biopsy if not stated otherwise.

*Differences among three patient groups were assessed using ANOVA with Tukey’s HSD test. *p* values for comparisons between patients with NASH-like lesions versus NAFL-like lesions are shown.

RA, rheumatoid arthritis; NASH, nonalcoholic steatohepatitis; NAFL, nonalcoholic fatty liver; IFH, interface hepatitis; NAFLD, nonalcoholic fatty liver disease; HRCT, high-resolution computed tomography; L/S ratio, liver-to-spleen attenuation ratio; NAS, NAFLD Activity Score; ALT, alanine aminotransferase; AST, aspartate aminotransferase; MTX, methotrexate; SE, standard error; ANOVA, analysis of variance

### Characteristics of RA patients with NASH- or NAFL-like pattern

As shown in Table 4, mean L/S ratios on HRCT scans at the time of liver biopsy were significantly different between NASH-like patients and NAFL-like patients (0.96 vs. 1.19, *p* = 0.018), which showed that the former group had more severe fatty deposition in the liver compared with the latter group. Data on L/S ratios prior to starting MTX therapy were available in approximately half of the patients in both groups (0.97 in NASH-like patients and 1.18 in NAFL-like patients). The data suggested that in NASH-like patients, fatty liver with fatty deposition > 30% had already existed at the time of introducing MTX treatment. In the NAS system, the disease activity grades for steatosis, lobular inflammation, and centrilobular ballooning were higher in NASH-like patients compared with NAFL-like patients (NAS, 3.9 vs. 0.1). The fibrosis stage was also more advanced in the former patients than the latter patients (stage ≥ 3, 59.1% vs. 14.3%). Antinuclear antibody was present in 72.7% of NASH-like patients and 57.1% of NAFL-like patients. One NASH-like patient was positive for anti-mitochondrial antibody M2. They did not, however, fulfill the diagnostic criteria for autoimmune hepatitis [37] or primary biliary cholangitis [38]. Similar levels of transaminases were observed in both patient groups. In addition, there were no significant differences in age, RA duration, BMI, serum levels of LDL-C or uric acid, or rates of hypertension or type 2 diabetes between these groups.

Regarding MTX use at the time of liver biopsy, there were no significant differences in means of cumulative dose (2.34 vs. 2.39 g) or duration (46.5 vs. 47.3 months) between NASH-like patients and NAFL-like patients. In addition, there was no significant difference in rates of steroid users or mean cumulative dose of steroids between the two groups.

## Discussion

In this study, the development of persistent transaminitis was observed in 51 out of 846 RA patients during MTX treatment. According to the multivariate regression analysis, obesity, type 2 diabetes, hypercholesterolemia, and hyperuricemia were significant factors associated with the development of persistent transaminitis. There was no significant difference in the mean cumulative dose of MTX. Among the 51 patients with persistent transaminitis, 42 showed fatty liver at US examinations. These patients had no evidence of alcoholic fatty liver, chronic viral hepatitis, autoimmune liver diseases, or hereditary liver diseases. Biopsy specimens were obtained from 32 patients, and the NASH-like pattern and NAFL-like pattern were found in 22 and 7 patients, respectively. There was no significant difference in means of exposure duration and cumulative dose of MTX between the two groups. Advanced stages of fibrosis were seen in 15 patients.

Obesity and type 2 diabetes have been reported to exert an influence on the development of liver injury during MTX treatment, especially fibrosis, in psoriasis patients [20–23]. As for RA patients, Kent et al. identified obesity and hyperlipidemia as risk factors for the permanent discontinuation of MTX because of AST elevations [39]. Other studies showed that elevations of hepatic aminotransferases were more likely to occur in MTX users with obesity and hyperlipidemia [40, 41]. In the present study, we identified obesity, type 2 diabetes, hyperlipidemia, and hyperuricemia as possible risk factors for persistent transaminitis during low-dose MTX treatment for RA. These factors are well recognized as risk factors associated with NAFLD [12, 17–19]. Dawwas and Aithal reported that compared with individuals with other liver diseases, patients with end-stage MTX-related liver diseases had a similar risk factor profile to those with NASH requiring liver transplantation [42]. In fact, we showed that more than 42 (82%) out of 51 patients of patients with persistent transaminitis had fatty liver at US examinations. We also found that the NASH-like pattern was the most prevalent histological pattern in liver biopsy samples. The similarity of risk factor profiles and histological findings between NAFLD/NASH and liver injury during MTX treatment may support the notion that these two entities share a common pathogenesis in RA patients.

Individuals susceptible to liver injury during MTX treatment may have underlying NAFLD/NASH. In the present study, all participants had normal ALT/AST levels prior to first starting MTX treatment, but the L/S ratios on HRCT scans at that time suggested the presence of underlying fatty liver in patients who developed NASH-like lesions during MTX treatment. Furthermore, the nine patients with severe transaminitis discontinued MTX during the follow-up period, but enzyme levels continued to be above the ULN. The exacerbation of preexisting NAFLD may be an important mechanism of liver injury observed in MTX-treated RA patients. Using data from a large cohort of RA and psoriatic arthritis patients initiating DMARDs, Curtis et al. indicated that transaminase elevations were 2.8-fold more likely in psoriatic arthritis patients than RA patients [43]. This may be explained by a higher incidence of NAFLD in psoriasis patients receiving systemic therapy compared with RA patients on such therapy [44].

It is important to evaluate histological changes during MTX treatment, especially the degree of hepatic fibrosis, because NAFLD patients with advanced stages of fibrosis is are at high risk for liver-related mortality [12–16]. Through liver biopsies, we found fibrosis in 24 (2.8%) out of 846 RA patients with a mean cumulative MTX dose of 2.48 g. Advanced fibrosis (≥ stage 3 in the Brunt criteria and the NAS system) was present in 15 patients (1.8%). No patients showed cirrhosis in liver biopsies. In a case-control study with 1571 MTX-treated patients with inflammatory arthritis, Quintin et al. performed liver biopsy on 41 patients who had elevated liver enzymes (mean cumulative MTX dose of 1.3 g). Fibrosis was detected in 22 patients (1.4%), but in most cases, it was mild [45]. In a systemic literature review, Visser and van der Heijde showed that after 4 years of MTX use with a mean cumulative MTX dose of 2.4 g, mild fibrosis (grade IIIA in the Roenigk scale [46]), severe fibrosis (grade IIIB), and cirrhosis (grade IV) were present in 15.3%, 1.3%, and 0.5% of biopsy specimens from RA patients, respectively. Pre-MTX biopsies, however, showed that mild fibrosis, severe fibrosis, and cirrhosis were preexisting in 9%, 0.3%, and 0.3% of RA patients [7]. By performing histological analysis of liver biopsy specimens from 42 RA patients, Ros et al. showed that 14% presented mild fibrosis before treatment with low-dose MTX, but after 4 years, no histological progression was observed [47]. These findings suggested that the development of advanced or severe hepatic fibrosis is uncommon during low-dose MTX treatment for RA. Similar data were reported in psoriasis patients [22, 48].

In this study, we monitored liver injury during MTX treatment through serial measurement of serum ALT and AST levels according to major rheumatology guidelines for the use of MTX in daily clinical practice for RA [27]. Previous studies reported that serial abnormal AST tests are significantly correlated with histological staging of liver injury in RA patients receiving MTX [49, 50]. Recent studies with NAFLD patients have shown that the usage of ALT and AST levels is not appropriate to screen for the presence of hepatic fibrosis, however, because transaminase levels are often normal in patients with such histological changes [51–54]. Therefore, we cannot exclude the possibility that some MTX-treated patients without persistent transaminitis in this study could have developed hepatic fibrosis during the treatment. Although liver biopsy is currently the most reliable approach for identifying the presence of hepatic fibrosis, routine surveillance biopsies are not recommended for RA patients receiving traditional doses of MTX because of cost, sampling errors, or procedure-related morbidity or mortality [6]. US evaluation of chronic liver disease such as hepatic fibrosis and cirrhosis is a reliable and effective alternative to pathological diagnosis [55, 56]. Conventional US is also the most common imaging technique to detect fatty liver. In NAFLD patients, however, this modality cannot grade hepatic fibrosis and cannot differentiate necro-inflammation and fibrosis (components of NASH) from simple steatosis [57, 58]. Considering the histological similarity between NAFLD/NASH and liver injury in RA patients receiving MTX treatment, conventional US may not be an appropriate tool for monitoring hepatic fibrosis during this treatment. Transient elastrography (TE), a recently developed non-invasive technique based on US monitoring of liver stiffness, has shown promising results for assessing the severity of hepatic fibrosis in non-obese patients with NAFLD [59, 60]. TE may be useful as a monitoring tool for hepatic fibrosis during low-dose MTX treatment of inflammatory arthritis such as RA [61].

Our findings are subject to several limitations. First, this study was not for patients who first began MTX treatment at enrollment. Therefore, we may have missed patients who discontinued MTX before the enrollment. Persistent transaminitis justifying liver biopsy is, however, considered a relatively rare event in the first year of MTX treatment for RA [41]. Thus, if we focused on the incident MTX cohort, we could not have obtained enough liver biopsies to evaluate the incidence of NASH-like lesions in liver injury occurring during MTX treatment for RA patients. Second, we did not examine biopsy specimens before the introduction of MTX or from patients without persistent transaminitis. These data would be useful to explore whether there may be a causative relationship between NASH and liver injury during MTX treatment. For ethical reasons, however, it was impossible to perform liver biopsy for patients without any signs or symptoms of liver disease. Finally, we did not include MTX-untreated RA patients as controls in this study. To examine the role that low-dose MTX can play in the development of persistent transaminitis in patients with NAFLD risk factors, it would be necessary to perform a prospective follow-up study including MTX-untreated controls. However, it was ethically unacceptable, because current guidelines recommend that MTX be part of the first treatment strategy in patients with active RA [1].

## Conclusions

Persistent transaminitis during low-dose MTX treatment is more likely to occur in RA patients with NAFLD risk factors, such as obesity, type 2 diabetes, hypercholesterolemia, and hyperuricemia. The NASH-like pattern is the most prevalent histological abnormality in RA patients with persistent transaminitis. Cumulative dose and duration seem unlikely to influence the severity of pathological findings. These findings suggest the strong association between NAFLD/NASH and liver injury observed in MTX-treated RA patients. NAFLD/NASH may predispose to the development of persistent transaminitis in RA patients during MTX treatment. The prevalence of NAFLD risk factors is rising in the general population and probably in RA patients and, consequently, the risk of liver injury associated with MTX treatment may also increase. The results of this study demonstrate the need for monitoring liver injury in RA patients with NAFLD risk factors during low-dose MTX treatment.
